# Biosynthesis and Mathematical Interpretation of Zero-Valent Iron NPs Using *Nigella sativa* Seed Tincture for Indemnification of Carcinogenic Metals Present in Industrial Effluents

**DOI:** 10.3390/molecules28083299

**Published:** 2023-04-07

**Authors:** Muhammad Naveed, Syeda Izma Makhdoom, Shafiq ur Rehman, Tariq Aziz, Farzana Bashir, Urooj Ali, Metab Alharbi, Abdulrahman Alshammari, Abdullah F. Alasmari

**Affiliations:** 1 Department of Biotechnology, Faculty of Science and Technology, University of Central Punjab, Lahore 54590, Pakistan; 2 Department of Basic and Applied Chemistry, Faculty of Sciences, University of Central Punjab, Lahore 54000, Pakistan; 3 Department of Agriculture, University of Ioaninna, 47100 Arta, Greece; 4 Principal Scientific Officer, Centre for Environmental Protection Studies, Pakistan Council of Scientific & Industrial Research Laboratory Complex, Lahore 54000, Pakistan; 5 Department of Biotechnology, Quaid-I-Azam University, Islamabad 45320, Pakistan; 6 Department of Pharmacology and Toxicology, College of Pharmacy, King Saud University, P.O. Box 2455, Riyadh 11451, Saudi Arabia

**Keywords:** zero-valent iron nanoparticles, indemnification, heavy metals, antioxidant, anti-inflammatory, anti-proliferative

## Abstract

Zero-valent iron nanoparticles (ZVI-NPs) are utilized for the indemnification of a wide range of environmental pollutants. Among the pollutants, heavy metal contamination is the major environmental concern due to their increasing prevalence and durability. In this study, heavy metal remediation capabilities are determined by the green synthesis of ZVI-NPs using aqueous seed extract of *Nigella sativa* which is a convenient, environmentally friendly, efficient, and cost-effective technique. The seed extract of *Nigella sativa* was utilized as a capping and reducing agent for the generation of ZVI-NPs. UV-visible spectrophotometry (UV-vis), scanning electron microscopy coupled with energy-dispersive X-ray spectroscopy (SEM-EDX), and Fourier transform infrared spectroscopy (FTIR) was used to investigate the ZVI-NP composition, shape, elemental constitution, and perspective functional groups, respectively. The biosynthesized ZVI-NPs displayed a peak of plasmon resonance spectra at 340 nm. The synthesized NPs were cylindrical in shape, with a size of 2 nm and (-OH) hydroxyl, (C-H) alkanes and alkynes N-C, N=C, C-O, =CH functional groups attached to the surface of ZVI-NPs. Heavy metals were successfully remediated from industrial wastewater collected from the various tanneries of Kasur. During the reaction duration of 24 h, different concentrations of ZVI-NPs (10 μg, 20 μg and 30 μg) per 100 mL were utilized for the removal of heavy metals from industrial wastewater. The 30 μg/100 mL of ZVI-NPs proved the pre-eminent concentration of NPs as it removed >90% of heavy metals. The synthesized ZVI-NPs were analyzed for compatibility with the biological system resulting in 87.7% free radical scavenging, 96.16% inhibition of protein denaturation, 60.29% and 46.13% anti-cancerism against U87-MG and HEK 293 cell lines, respectively. The physiochemical and exposure mathematical models of ZVI-NPs represented them as stable and ecofriendly NPs. It proved that biologically synthesized NPs from a seed tincture of *Nigella sativa* have a strong potential to indemnify heavy metals found in industrial effluent samples.

## 1. Introduction

Amongst the latest science and technology fields one can say that nanotechnology is the latest field of innovative research in this era of science and technology. The applications of nanotechnology are concerned with various modern technological aspects, thus the products formed as a result of nanotechnology are very efficient, more functional, economical, and smarter to use as compared to the products formed through previous conventional technologies [1]. Nanoparticles (NPs) as a result of nanotechnological products usually range from 1 nm to 100 nm in size, and cannot be visualized by the naked human eye. Structurally, NPs consist of three main parts, viz (i). surface of the nanoparticle, (ii). shell of the nanoparticle, and (iii). core of the nanoparticle [2]. The surface layer is made up of polymer used for the synthesis of nanoparticles to which various functional groups are attached. The shell of the nanoparticle is made up of different material as compared to the core which is the central point of the synthesized nanoparticle [3].

Due to recent globalization, industrialization, and various anthropogenic activities, the contamination of soil and water is increasing day by day and because of this developing countries are suffering from various problems such as heavy metal pollution which is one of the most widespread environmental problems of the modern world as its hazardous effects are present in all ecosystems [4,5,6,7]. Heavy metals are those toxic elements which contaminate the water in developing countries due to their excessive discharge from industrial zones and they are non-biodegradable and persistent in nature [8]. Industrial wastewater contains heavy metals like cadmium, lead, arsenic, mercury, and chromium-IV in high concentrations that cause multiple lethal diseases in living beings, when discharged untreated into drinking water bodies [9]. Heavy metals in industrial wastewater have acute to chronic toxic effects on the health of human beings. Prolonged exposure of a biological system to heavy metals can causes muscular and neurological degenerative processes, gastrointestinal, kidney and immune system dysfunction, skin lesions, vascular damage, and cancer [10].

Zero-valent iron NPs (ZVI-NPs) reveal pre-eminent magnetic ability regarding environmental cleanup by remediating various pollutants especially heavy metals [11,12,13]. Several studies have reported ZVI as a reducing agent for the remediation treatment of halogenated hydrocarbon pollutants in the environment for both contaminated soil and water sources [14,15]. The core of ZVI-NPs is iron that causes the heavy metal to be attracted and attached within its core thus removing the metal contaminant completely from the wastewater. The size and shape of ZVI-NPs can significantly impact their reactivity, transport, stability, and toxicity in environmental remediation applications. It is important to carefully consider these factors when designing and using ZVI-NPs for pollution control and other environmental applications [16].

The capacity of ZVI-NPs to remediate heavy-metal-contaminated wastewater effluents from various sectors has been extensively explored. Because of their low toxicity and relative abundance in nature, ZVI-NPs have found widespread use in the field of pollution abatement. ZVI-NPs have a Fe (0) core and a Fe oxide outer shell, with the core capable of reducing and the outside shell serving as a reaction site for chemisorption and electrostatic interactions [17]. The removal of heavy metals from aqueous phases using ZVI-NPs has been aided by mechanisms such as reduction, absorption, precipitation, and mineralization. ZVI-NPs have ligand-like characteristics when dissolved in water. The iron oxide shell is positively charged at low pH, attracting anions such as phosphates and sulphates, and negatively charged at high pH, attracting cations such as metal ions. After the reaction, heavy metals are sequestered on the solid ZVI-NP surface. Surface plasmon resonance (SPR) is a phenomenon that can occur in ZVI-NPs when they are exposed to electromagnetic radiation, such as light. When light interacts with ZVI-NPs, it can excite the free electrons on the surface of the nanoparticles, causing them to oscillate at a particular frequency. This frequency corresponds to the resonant frequency of the surface plasmon, which can be detected and measured as a change in the refractive index of the surrounding medium [18].

*Nigella sativa,* also known as cumin seed, is a medicinal herb which is 20–30 cm tall, and whose leaves are linear and finely divided. The flowers of *Nigella sativa* consist of 5 to 10 petals which are blue to white in coloration. The flower of *Nigella sativa* have a large capsule with three to seven follicle-containing seeds in its sac [19]. *Nigella sativa* contains multiple biopolymers along with antioxidant and anti-inflammatory compounds. During the biological synthesis of NPs using *Nigella sativa* seed tincture, the compounds present in the extract serve as a capping, reducing and stabilizing agent to the synthesized NPs [20]. The current study reveals a cost effective and efficient method for the remediation of heavy metals from industrial effluents at laboratory scale by utilizing nanotechnology. The biosynthesis of ZVI-NPs from the seed extract of *Nigella sativa* at optimized parameters was mathematically modelled against various physiochemical and exposure parameters and tested against the removal of carcinogenic heavy metals from industrial effluents. Further, the functionality, size and shape of ZVI-NPs is characterized by UV-Visible spectrophotometry, SEM, EDX, and FTIR. Moreover, the synthesized ZVI-NPs were investigated for their side effects on biological systems by performing antioxidant, anti-inflammatory, and cytotoxicity activities.

## 2. Results

### 2.1. Detection of Heavy Metals in Collected Wastewater Samples

The collected wastewater samples from five different tannery industries of Kasur were named as S-1, S-2, S-3, S-4, and S-5. The collected industrial wastewater samples were capped properly. Heavy metals were detected in the collected industrial wastewater samples. Initial concentration in ppm of the detected heavy metals analyzed by atomic absorption spectroscopy is mentioned in Table 1.

### 2.2. Phytochemical Screening of Nigella sativa Seed Extract

The screened phytochemicals of seed extract of *Nigella sativa* are summarized in Table 2.

### 2.3. Determination of Reducing Power of Nigella sativa Extract

**Figure 1:**
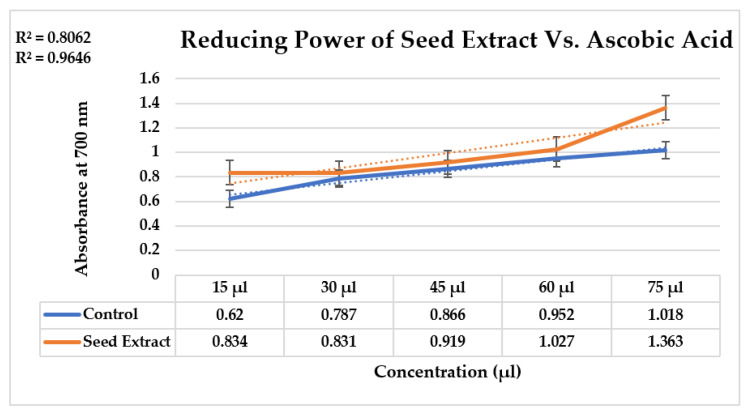
Determined reducing power of seed extract with control, i.e., ascorbic acid, showing the strong relationship, thus showing the capability of seed extract to reduce the ZVI-NPs.

Reducing power assay is based on the reduction mechanism of a substance, that has a potential to reduce potassium ferricyanide to potassium ferrocyanide, which further reacts with the ferric chloride to form ferric–ferrous complex that gives maximum absorbance when measured at 700 nm. The reducing power of seed extract of *Nigella sativa* (R^2^ = 0.9646) increases with the increase in its concentration, thus showing a good linear relation of reducing power as compared to the control, i.e., ascorbic acid (R^2^ = 0.8062) (Figure 1).

### 2.4. Biosynthesis of ZVI-NPs

**Figure 2:**
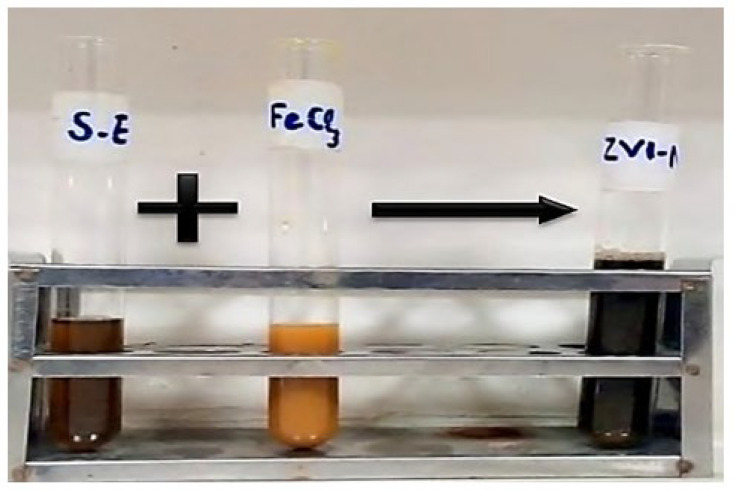
Observable color change of FeCl_3_ precursor from orange color to black color by the addition of *Nigella sativa* seed extract indicating the synthesis of ZVI-NPs.

The addition of seed extract of *Nigella sativa* reduced the Fe^3+^ to Fe^0^ of the precursor FeCl_3_. A blackish brown color change was observed on the dropwise addition of seed extract to the precursor (Figure 2). The increase in the incubation time increased the reduction of Fe^3+^ ions into iron particles with zero valency, thus shows the formation of ZVI-NPs.

### 2.5. Characterization of ZVI-NPs

#### 2.5.1. UV-Visible Spectrophotometry

**Figure 3:**
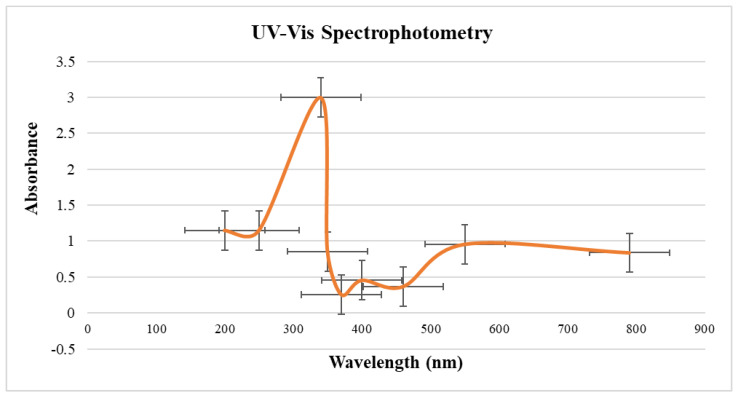
UV visible spectroscopy for ZVI-NPs synthesized using *Nigella sativa* seed extract synthesized from 25 mM precursor solution of FeCl_3_.

SPR occurs when light is absorbed by the ZVI-NPs, causing the electrons to vibrate at a specific frequency, which results in a change in the reflectivity or absorption of the incident light. This can be detected by monitoring the changes in the intensity of the reflected or transmitted light, allowing for the quantification of the surface properties of the nanoparticles, such as their size, shape, and surface chemistry. The maximum absorbance by ZVI-NPs was recorded at 340 nm of UV-Vis spectra corresponding to the synthesis of ZVINPs, that increases with the increase in the time of incubation (Figure 3). The observable spectrum shows the reduction of Fe^3+^ to Fe^0^, thus indicating the synthesis of ZVI-NPs.

Theorem-type environments (including propositions, lemmas, corollaries etc.) can be formatted as follows:

#### 2.5.2. Scanning Electron Microscopy (SEM)

**Figure 4:**
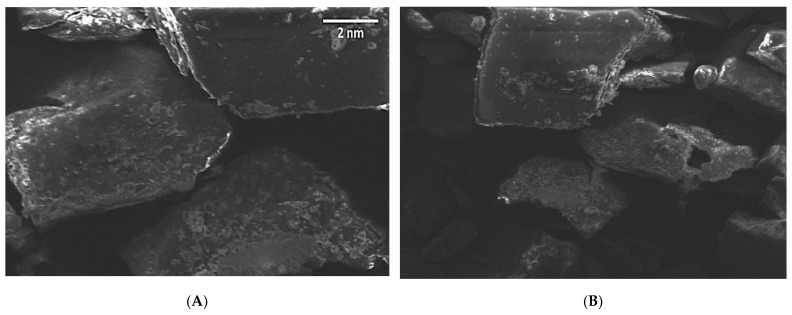
Scanning electron micrograph of ZVI-NPs synthesized by seed extract of *Nigella sativa* at concentration of 25 mM of FeCl_3_. (**A**). at 1000×. (**B**). at 500×.

ZVI-NPs were well disseminated and uniform in size, i.e., cylindrical in shape, according to a typical electron micrograph. Furthermore, Image J software revealed that the average crystalline size of 25 mM ZVI-NPs is 2 nm (Figure 4).

#### 2.5.3. Fourier Transform Infrared Spectroscopy (FTIR)

**Figure 5:**
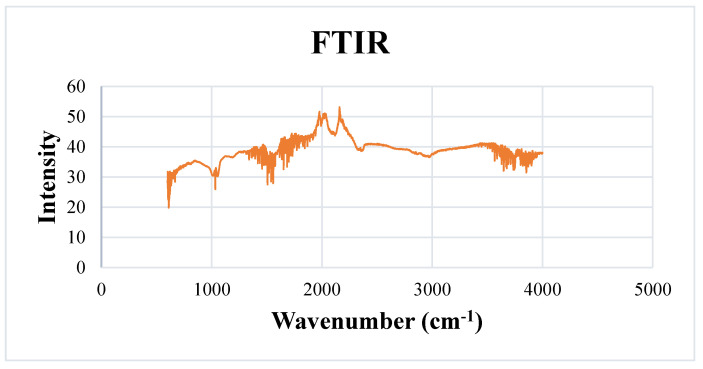
FTIR analysis of ZVI-NPs showing capping and reducing functional groups of seed extract *Nigella sativa* thus stabilizing the synthesized NPs.

The surface structures and functional characteristic groups responsible for the reduction and stabilization of green-synthesized ZVI-NPs are revealed by FTIR spectra (Figure 5). The peak 2914.8 cm^−1^ characterizes the O–H stretching vibrational region of ZVI-NPs. The peak 2847.7 cm^−1^ identifies the functional groups of alkanes and aliphatic hydrocarbons. The peaks 1999.3 cm^−1^, 1993.2 cm^−1^, and 1912.6 cm^−1^ characterize the C ═ O stretching vibrations of the carbonyl group of seed extract. The peak 1507.7 cm^−1^ is associated with the presence of aromatic compounds in the seed extract. The peaks 1194.6 cm^−1^ and 1012.4 cm^−1^ confirm the presence of symmetrical and asymmetrical C–O vibrational stretches. The peaks characterized by FTIR show all the active functional groups that are part of the seed extract used and are present on the surface of ZVI-NPs as an efficient capping and reducing agent.

#### 2.5.4. Energy-Dispersive X-ray Spectroscopy (EDX)

**Figure 6:**
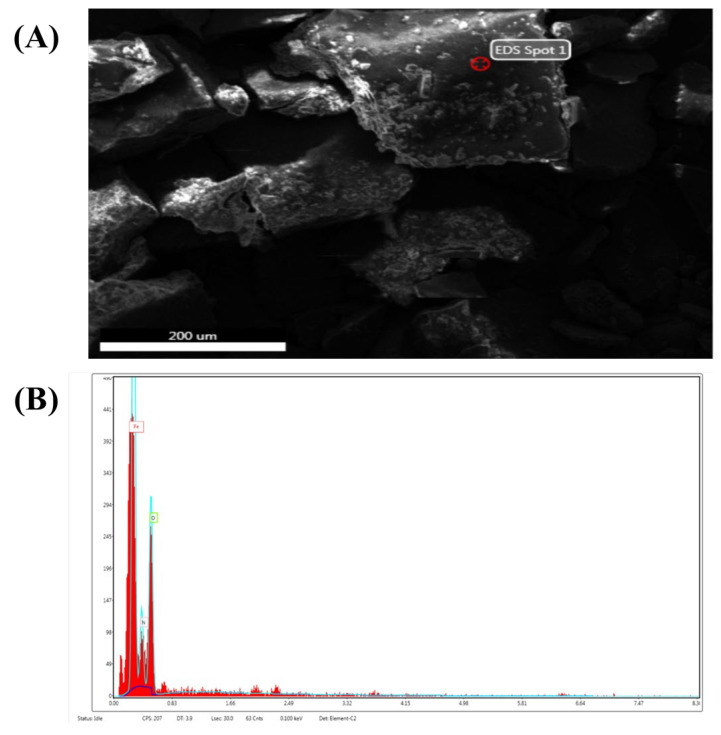
(**A**) Showing EDX spot of which elemental composition is determined. (**B**) Graphical representation of detected elements.

The EDX confirmed the elemental composition of the synthesized ZVI-NPs. It confirms that ZVI-NPs are made up of iron (43.02%), nitrogen (21.94%), and oxygen (35.04%) (Figure 6A,B).

### 2.6. Remediation of Heavy Metals in Wastewater by ZVI-NPs

After treating each industrial wastewater sample with ZVI-NPs, various concentrations of heavy metals were recorded after the interval of 60 min (Table 3).

**Figure 7:**
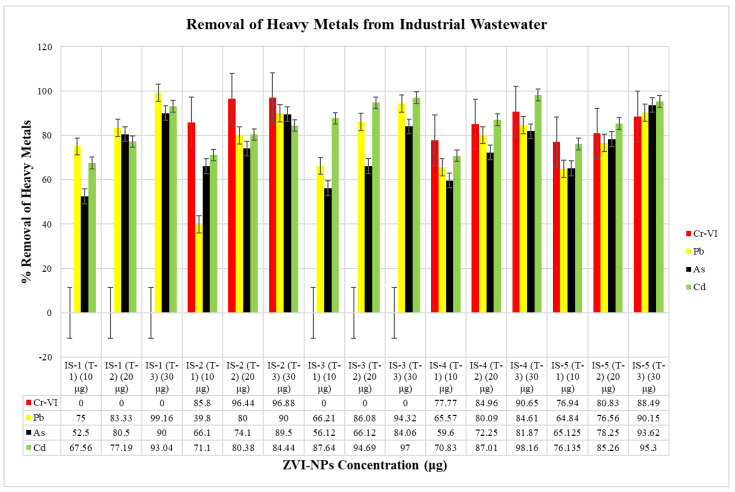
Graphical representation of the remediated heavy metals (Cr-VI, Pb, As, and Pb) from 100 mL of industrial wastewater at respective concentrations of ZVI-NPs (10 μg, 20 μg, and 30 μg).

There was a significant decrease in the concentration of heavy metals in the treated wastewater samples. The percentage removal of heavy metals was recorded to be above 90% at 30 μg concentration of ZVI-NPs (Figure 7). Thus, it was interpreted that the 30 μg concentration of ZVI-NPs is effective in removal of heavy metals (Cr-VI, Pb, As, and Cd) from 100 mL of industrial wastewater.

### 2.7. Anti-Inflammatory Analysis of ZVI-NPs

**Figure 8:**
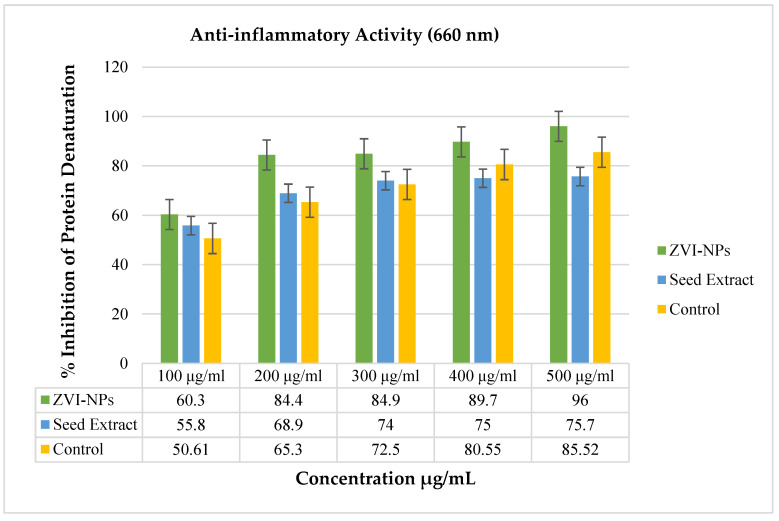
Graphical presentation of % inhibition of protein denaturation at specific concentrations of control (aspirin), seed extract, and ZVI-NPs, thus showing 96% anti-inflammatory activity by ZVI-NPs at 500 μg/mL.

The maximum percentage inhibition of protein denaturation was 96% and 75.7%, observed at 500 μg/mL concentration of ZVI-NPs and seed extract of *Nigella sativa*, respectively. Statistical analysis has shown that all the results of anti-inflammatory tests have a significant relationship (*p* < 0.01) when compared with the control, i.e., aspirin (Table 4). The analyzed results can be interpreted to show that the synthesized ZVI-NPs are more effective at inhibiting the denaturation of albumin protein as compared to seed extract of *Nigella sativa* and control drug (aspirin) (Figure 8).

### 2.8. Antioxidant Analysis

**Figure 9:**
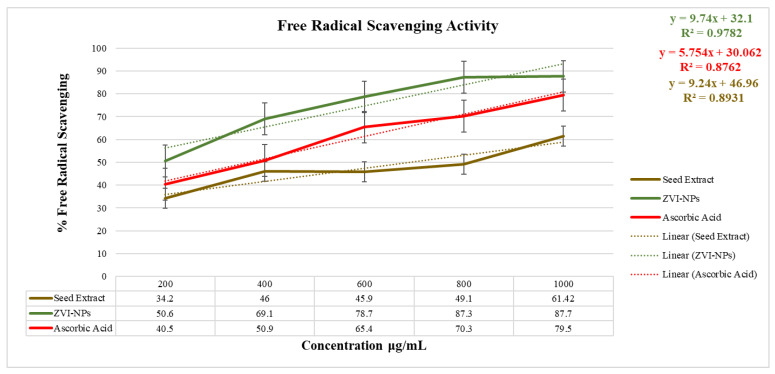
Graphical representation for the calculation of IC50 and antioxidant activity showing the highest scavenging percentage of ZVI-NPs (87.7%) at the concentration of 1000 μg/mL.

The ability of the DPPH radical to reduce was assessed by the decrease in its absorbance at 517 nm caused by several antioxidants, i.e., relationship between antioxidant compounds and radical advance; the scavenging of the radical via hydrogen donation is induced by the decrease in absorbance of the DPPH radical caused by antioxidants. Table 5 shows the percentage of free radical scavenging activity and IC50 of standard (ascorbic acid), seed extract, and ZVI-NPs of which NPs showed the highest antioxidant activity of 87.7% at a maximum concentration of 1000 μg/mL. The IC50 value was calculated by graphical analysis (Figure 9) in which ZVI-NPs showed the lowest IC50 value (194.77) as compared to the standard, i.e., ascorbic acid (596.7) and seed extract (387.56) which proves that the synthesized ZVI-NPs are more potent for a biological system in contrast to the standard and seed extract (Table 5).

### 2.9. Cytotoxicity Testing of ZVI-NPs

**Figure 10:**
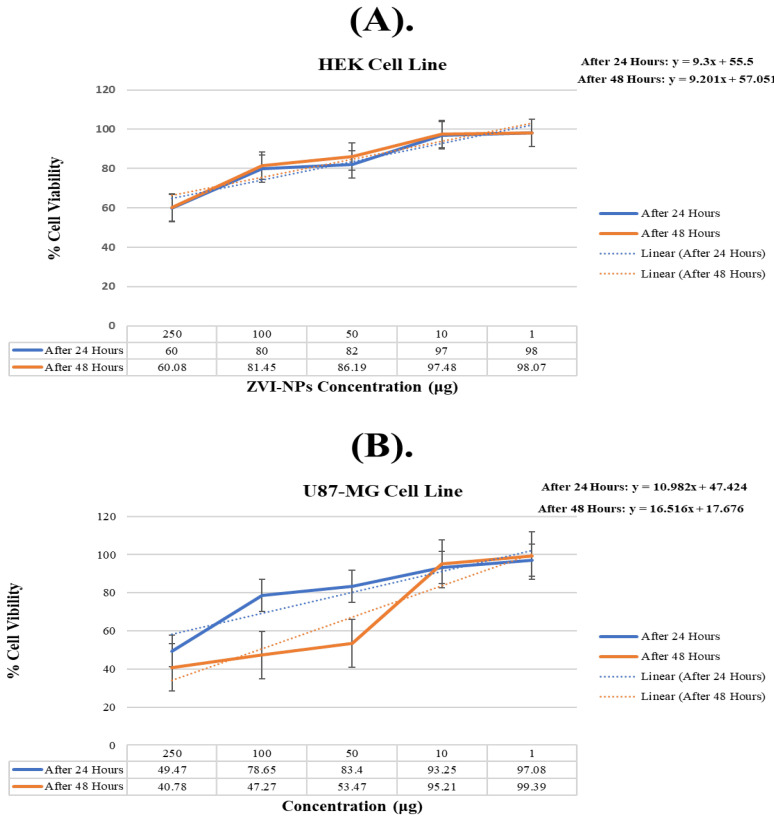
Graphical presentation of % cell viability. (**A**). U87-MG cell line (**B**). HEK cell lines.

Cytotoxicity testing of ZVI-NPs gave pre-eminent results against U87-MG and HEK cell lines. The increased concentration of ZVI-NPs causes the decrease in the viability of U87-MG (Figure 10A) and HEK cells (Figure 10B) along with the IC50 value of used NPs thus enhancing the potency of ZVI-NPs with the biological system (Table 6). There were significant morphological changes in U87-MG and HEK cells that were also observed and compared with the positive control, solvent control, and MTT-based treated cells (Table 7).

### 2.10. Mathematical Models

#### 2.10.1. Density Parameter

**Figure 11:**
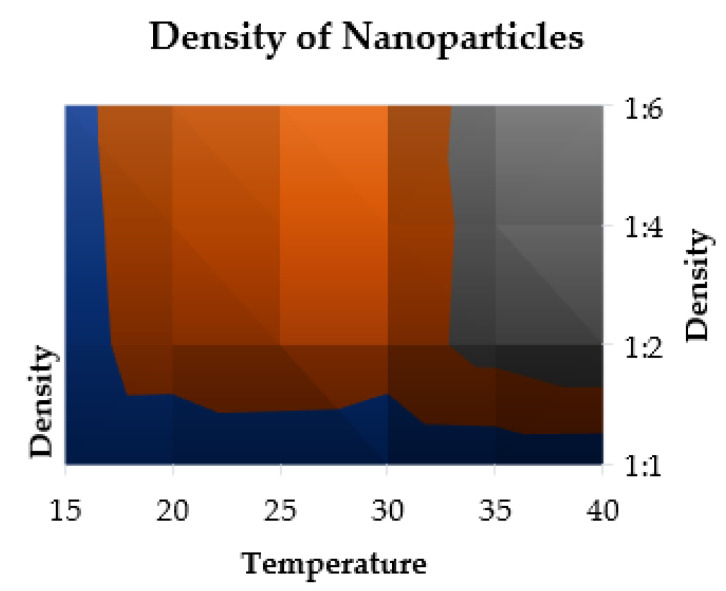
Density profiling of zero-valent iron NPs at the ratio 1:1, 1:2, 1:4, and 1:6 of the water, showing the distribution of ZVI-NP densities within a wastewater at varying temperature and water ratios.

The density of ZVI-NPs changes with the change in temperature and solvent accessibility ratio (Table 8). The mathematical model of density is represented in Figure 11 showing the increase in density value of ZVI-NPs with the increase in temperature and solvent.

#### 2.10.2. Specific Heat Capacity

**Figure 12:**
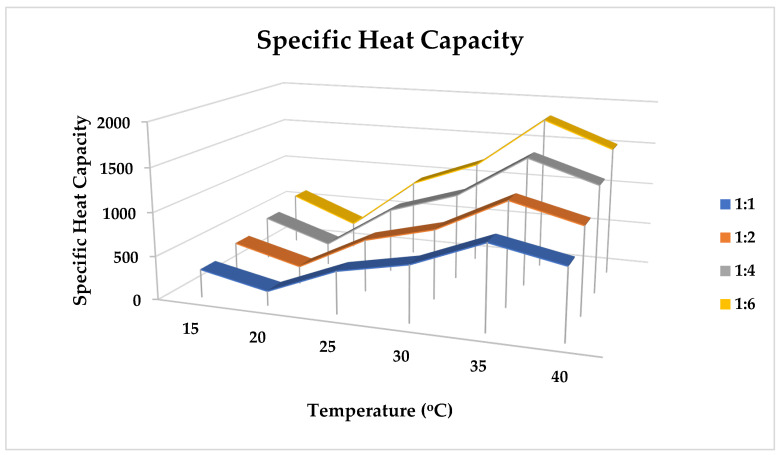
Mathematical model of specific heat capacity of ZVI-NPs at a particular temperature range of (15–40 °C) along with the solvent mixing at 1:1, 1:2, 1:4, and 1:6.

ZVI-NPs’ specific heat capacity varies as a function of temperature and the ratio of solvent accessibility (Table 9). Figure 12 illustrates the mathematical representation of heat capacity and the rise in ZVI-NPs’ specific heat capacity value with temperature and solvent.

#### 2.10.3. Thermal Conductivity via Heat Transfer

**Figure 13:**
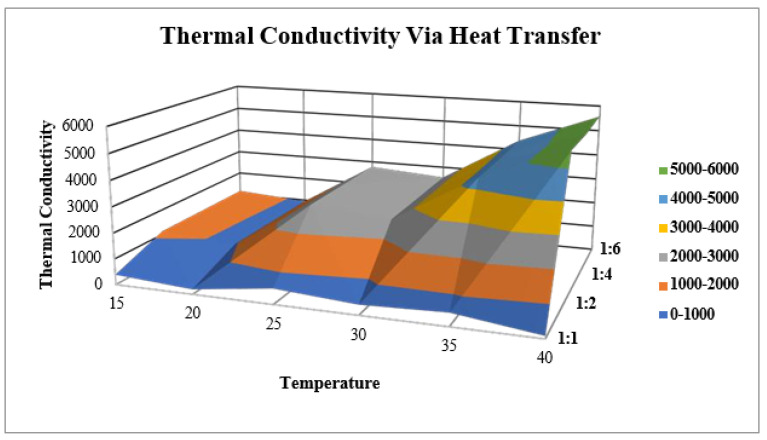
Mathematical model of thermal conductivity of ZVI-NPs at a particular temperature range of (15–40 °C) along with the solvent mixing at 1:1, 1:2, 1:4, and 1:6.

Table 10 and Figure 13 shows the relationship between temperature and solvent on ZVI-NPs’ thermal conductivity values as well as the mathematical representation of thermal conductivity.

#### 2.10.4. Worker’s Model

**Figure 14:**
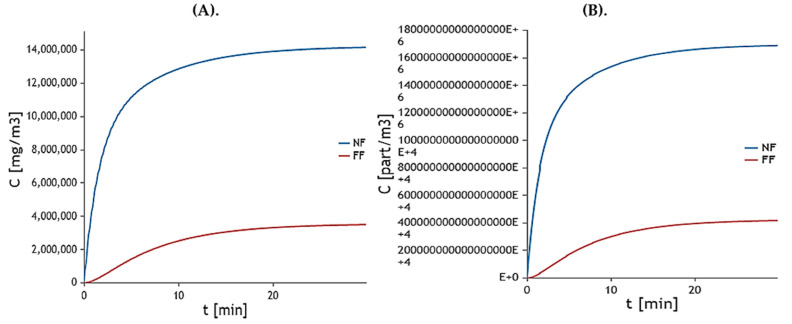
Exposure model based on working parameters showing (**A**). Mass production of ZVI-NPs (at large scale) (**B**). Particle production of ZVI-NPs (at small scale) at a particular time.

The graphical representation of worker’s exposure model presents mass production of ZVI-NPs (Figure 14A) and small scale synthesis (Figure 14B). Both large and small scale production shows a controlled biological synthesis of ZVI-NPs near field (NF) and far field (FF).

#### 2.10.5. Environmental Model

**Figure 15:**
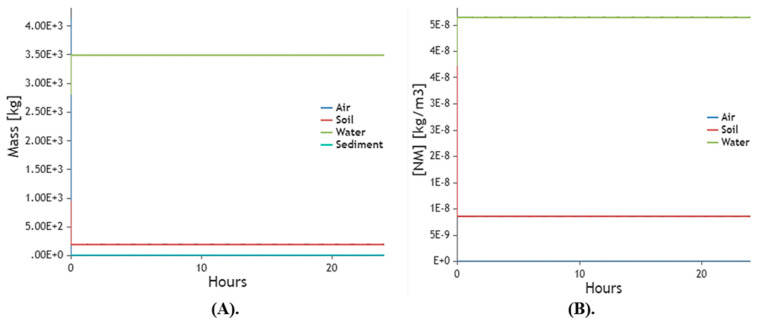
Based on environmental data, this exposure model displays (**A**). ZVI-NPs are being manufactured in large quantities (**B**). ZVI-NPs’ particle generation (on a small scale) at a specific moment.

The graphic representation of the worker exposure model shows small-scale synthesis (Figure 15A,B) and mass production of ZVI-NPs. In the air, soil, water, and sediment, both large- and small-scale production exhibited environmentally favorable behavior.

## 3. Materials and Methods

**Figure 16:**
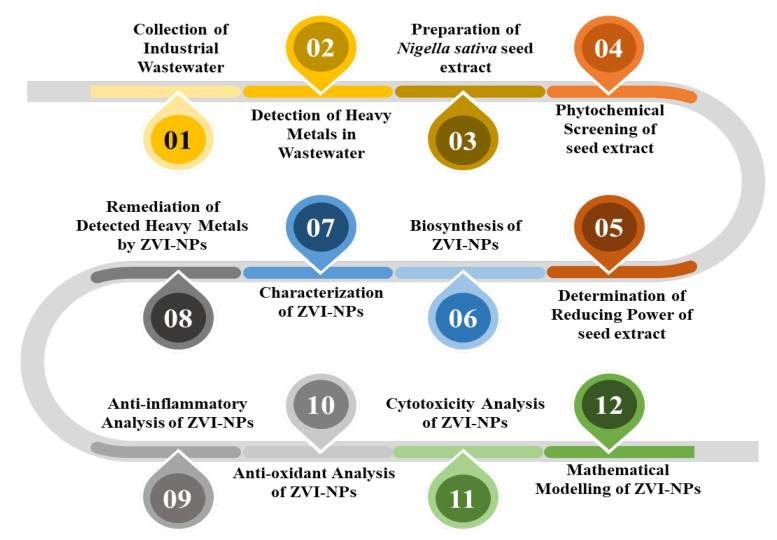
A schematic illustration of showing the overview of the methodology used to synthesize ZVI-NPs and its application.

A schematic illustration of methodology is shown in Figure 16.

### 3.1. Collection of Industrial Wastewater Samples

Collection of effluents was done from various tanneries in the industry of Kasur, Lahore district division, Punjab province, Pakistan. The samples were collected by directly filling the container with industrial wastewater. After the collection, samples were subjected to tests to detect heavy metal ions in industrial effluents.

### 3.2. Detection of Heavy Metals in Collected Wastewater Samples

The collected industrial wastewater was analyzed to detect five main carcinogenic heavy metals, namely, (i). chromium-IV (Cr-IV), (ii). arsenic (As), (iii). cadmium (Cd), (iv). mercury (Hg), and (v). lead (Pb) using atomic absorption spectroscopy (AAS). The initial concentration of detected heavy metals was also measured in parts per million (ppm) and was compared with the standard concentration of heavy metals recommended by the World Health Organization (WHO) [21] for industrial wastewater.

### 3.3. Preparation of Seed Extract of Nigella sativa

The seeds of *Nigella sativa* were purchased from nearest retailer’s shop Lahore, Pakistan. The seeds, weighed at 20 gm, were washed thoroughly with distilled water thrice and dried in a hot air oven. The dried seeds were ground in an electric grinder. In a 250 mL beaker, 100 mL of distilled water was added and 20 gm of the ground seed of *Nigella sativa*. The beaker was placed in an ultra-sonicator for 60 min at 80 °C to obtain the seed extract. The sonicated solution was filtered using Whatman filter paper No. 1 in a conical flask. The filtered solution was stored at 4 °C in a lab refrigerator to use further for the synthesis of ZVI-NPs.

### 3.4. Phytochemical Screening of Nigella Sativa Seed Extract

#### 3.4.1. Wagner’s Test

Wagner’s test was performed to screen alkaloids. For this reason, 2–3 mL of seed separate was stepped through in a test tube. A total of 1 mL of HCl and several drops of Wagner’s reagent were added to the seed tincture. The test tube was shaken well to obtain the reddish-brown color which demonstrates the presence of alkaloids.

#### 3.4.2. Foam Test

Foam test was performed to distinguish saponins in the extricated solution of *Nigella sativa* seeds. To this end, 5 mL of the seed tincture was taken through in a test tube, and 5 mL of refined water was added, and it was shaken vigorously. An arrangement of stable froth shows the presence of saponins.

#### 3.4.3. Ferric Chloride Test

Ferric chloride test was performed to separate phenols present in the seed tincture. A total of 5 mL of seed separate was taken in a test cylinder and several drops of impartial 5% ferric chloride solution was added. The seed concentrate gives a blue green tone with ferric chloride, demonstrating the presence of phenols.

#### 3.4.4. Braymer’s Test

Braymer’s test was performed to recognize the presence of tannins. It was performed by adding 2 mL of refined water and several drops of ferric chloride solution in 2 mL of seed tincture of *Nigella sativa*. The development of green precipitates demonstrates the presence of tannins.

#### 3.4.5. Salkowski’s Test

Salkowski’s test was performed to show terpenoids in the extricated seed tincture of *Nigella sativa*. For this, 2 mL of seed tincture was blended in with 2 mL of chloroform and 2 mL of concentrated H_2_SO_4_. Development of a yellow variety shows the presence of terpenoids in the seed extract.

#### 3.4.6. Bontrager’s Test

The phytochemical quinones was screened by carrying out Bontrager’s test. In 3 mL of seed tincture, 3 mL of chloroform was added. This frames a layer that isolates seed extricate from added chloroform. A total of 5% of potassium hydroxide was added to the layer framed. An event of a red variety in the layer framed demonstrates the presence of quinones.

#### 3.4.7. Keller–Killian’s Test

Keller–Killian’s test was performed to screen cardiac glycosides from arranged seed extract of *Nigella sativa*. A total of 2 mL of HCl, sodium nitroprusside and sodium hydroxide were added to 2 mL of seed extricate. Development of a pink to crimson variety demonstrates the presence of cardiac glycosides.

#### 3.4.8. Glycosides Test

The phytochemical glycosides were screened by performing glycosides test. To this end, 2 mL of seed tincture of *Nigella sativa* was blended in with 3 mL of chloroform and 10% of alkali solution. Occurrence of pink shading shows the presence of glycosides.

#### 3.4.9. Alkaline Reagent Test

Alkaline reagent test was performed to screen flavonoids from the seed tincture of *Nigella sativa*. NaOH was added to the 2 mL of seed extricate. Sign of yellow variety shows the presence of flavonoids.

#### 3.4.10. Precipitate Test

The screening of phytochemical phlobatannins was completed by performing the precipitate test. In 1 mL of seed extract of *Nigella sativa* a few drops of 2% HCl were added. Appearance of red hastens shows the presence of phlobatannins in the extracted seed tincture.

### 3.5. Determination of Reducing Power of Nigella sativa Extract

The reducing capacity of the seed extract of *Nigella sativa* was determined by the transition of Fe^3+^/ferricyanide complex to Fe^2+^/ferrocyanide complex.

#### 3.5.1. Preparation of Standard Solution

1 M of ascorbic acid was prepared as a standard solution to investigate the reducing power of the plant at different concentrations: 15, 30, 45, 60, and 75 μg/mL.

#### 3.5.2. Preparation of Test Sample

Previously prepared seed extract of *Nigella sativa* was utilized as a test sample at the same concentration as that of the standard solution, i.e., 15, 30, 45, 60, and 75 μg/mL.

#### 3.5.3. Protocol for Determination of Reducing Power

The pre-arranged norm and test samples were mixed with 2.5 mL of phosphate support (6.6 pH), 1% potassium ferricyanide (K_3_Fe (CN)_6_), and fluctuated centralizations of the two solutions. For 30 min, the blend was warmed in water at 60 °C. Trichloroacetic acid (TCA) was included 2.5 mL after the incubated solution had cooled. For 10 min, the liquid was centrifuged at 3000 rpm. A total of 0.5 mL of fresh ferric chloride solution (0.1%) and 2.5 mL of refined water were added to the top layer of the centrifuged arrangement. Utilizing an UV-Vis spectrophotometer, the arrangement’s absorbance at 700 nm was determined. During the absorbance investigation, refined water was utilized as a blank. An expansion in absorbance at 700 nm suggests that plant extract has a more prominent reducing capacity [22].

### 3.6. Biosynthesis of ZVI-NPs

ZVI-NPs were synthesized by mixing 25 mM of FeCl_3_ precursor with the seed extract of *Nigella sativa* in a fixed ratio of 1:9, respectively. The seed extract was mixed with FeCl_3_ precursor dropwise with continuous stirring on a magnetic stirrer at 1500 rpm in an aluminum closed chamber to avoid contact with light during the reaction. After complete addition of seed extract with FeCl_3_ in a fixed ratio, the solution was stirred for another 30 min, and was kept in an incubation for 24 h in the dark at room temperature. The incubated solution was then centrifuged at 4000 rpm for 30 min. The supernatant was discarded, and the pellet was washed with distilled water thrice. The washed pellet was poured into an evaporating dish and was dried in a hot air oven at 80 °C for 6–7 h. The dried pellet was stored in an Eppendorf tube at room temperature and was subjected to further analysis.

### 3.7. Characterization of ZVI-NPs

#### 3.7.1. UV-Visible Spectrophotometry

The presence of ZVI-NPs was confirmed using a UV visible spectrophotometer that monitored the surface plasmon resonance band (200–800 nm). According to Ananda Lakshmi et al. [23], this band correlates to the absorption of colloidal ZVI-NPs in the area (250–370 nm) due to the activation of surface plasmon vibration, confirming the presence of zero-valent iron NPs.

#### 3.7.2. Scanning Electron Microscopy (SEM)

SEM was used to determine the structure of the NPs that were obtained. At room temperature, dried samples of synthesized ZVI-NPs were placed on double-conductive tape fixed to a sample holder. The samples were coated with a platinum–gold coating to improve conductivity. Following that, samples were seen at a voltage of 12.50 kV.

#### 3.7.3. Fourier Transform Infrared Spectroscopy (FTIR)

The functional groups responsible for the production of ZVI-NPs were determined and the FTIR spectrum was recorded using a Fourier transform infrared spectrophotometer. For FTIR measurements, the solution of produced silver NPs was centrifuged for 30 min at 10,000 rpm.

#### 3.7.4. Energy-Dispersive X-ray Spectroscopy (EDX)

EDX was used to determine the composition of elements present on the surface of ZVI-NPs. To this end, specific X-rays were bombarded on to the sample. Resultantly, some radiations were emitted by the sample which were amplified and recorded. The recorded diffracted rays were measured to analyze the elemental composition and results were displayed on the screen.

### 3.8. Remediation of Heavy Metals in Wastewater by ZVI-NPs

Heavy metals that were detected previously (Section 3.2) were remediated by the ZVI-NPs synthesized formerly (Section 2.6). In different conical flasks all industrial wastewater samples were taken measuring 100 mL. ZVI-NPs were added in the wastewater samples at the concentrations of 10 μg, 20 μg, 30 μg and were placed in the incubator shaker for 60 min at 150 rpm. After the period of incubation, the ZVI-NPs were drawn out of the industrial wastewater samples by simply centrifuging out the added NPs. The treated wastewater samples were analyzed for the final concentration of heavy metals through atomic absorption spectroscopy (AAS). Percentage of the removal of the heavy metals was calculated by:

**% Removal of Heavy Metals = C_i_ − C_f_/C_i_ × 100**

where, C_i_ is initial concentration of heavy metals and C_f_ is the final concentration of heavy metals present in industrial wastewater.

### 3.9. Anti-Inflammatory Analysis

As per Ahmed et al., the mitigating impact of *Nigella sativa* seed extract and greenly created ZVI-NPs was examined using the protein denaturation technique [23]. Approximately 2.8 mL of phosphate–buffer saline solution (pH 6.4), 0.2 mL of hen’s egg white, and 2 mL of *Nigella sativa* seed extricate and biosynthesiz1ed ZVI-NPs at convergences of 100, 200, 300, 400, and 500 g/mL were mixed. The mixture blends were warmed to 70 °C for 5 min after being incubated at 37 °C for 20 min. The turbidity was evaluated at 660 nm with a UV-Vis spectrophotometer, after cooling. Similar convergences of the anti-inflammatory medicine (acetyl salicylic acid) drug were utilized as the control at 100, 200, 300, 400, and 500 g/mL. The anti-inflammatory analysis was performed in a triplicate manner and protein inhibition percentage was calculated by the following formula:

**% Inhibition of protein denaturation = 1 − A_s_/A_o_ × 100**

where, A_o_ is the absorbance of control and A_s_ is the absorbance of the test sample measured through UV-Visible spectroscopy at 660 nm.

### 3.10. Anti-Oxidant Analysis of ZVI-NPs

The free radical scavenging capacity of both seed extract of *Nigella sativa* and synthesized ZVI-NPs was analyzed using 2,2-diphenyl-1-picrylhydrazyl radical (DPPH)-based assay [15]. The seed extract and ZVI-NPs for the DPPH examination were analyzed at various convergences of 200, 400, 600, 800, and 1000 μL. At first, 0.1 mM of DPPH solution was prepared by combing 3.94 mg of it into 100 mL of methanol and saving in dim conditions for 30 °C at room temperature to keep the pre-arranged solution away from contact of light. A total of 0.1 mL of 0.1 nm DPPH was added to different groupings of prepared seed tincture and ZVI-NPs. The mixture was warmed for 30 min and the absorbance at 517 nm was measured against the positive control, i.e., DPPH. The free radical scavenging analysis was performed in a triplicate manner and the percentage of DPPH inhibition was determined by the following formula:

**% DPPH inhibition = A_o_ − A_s_/A_o_ × 100**

where, A_o_ is the absorbance of control and A_s_ is the absorbance of the test sample measured through UV-Visible spectroscopy at 517 nm.

### 3.11. Cytotoxicity Testing of ZVI-NPs

Cytotoxicity testing was performed on adherent cell lines, i.e., U87-MG and HEK. The cells were cultured in T-25 flasks in pre-warmed complete Dulbecco’s Modified Eagle Media (DMEM). The media was supplemented with 10% Fetal Bovine Serum and 1% of pen-strep was also added to cell culture medium to prevent contamination of micro-organisms. Subsequently, the culture flasks were kept in a humidified water-jacketed incubator at 37 °C temperature and 5% CO_2_ to allow for U87-MG and HEK cell growth. MTT assay was used to observe the anti-cancer activity of the synthesized ZVI-NPs at various concentrations through percent cell viability. To this end, the tetrazolium (component of yellow MTT) was cleaved into blue insoluble crystals of formazan by the viable cells. This reduction reaction is catalyzed by the active mitochondrial dehydrogenase enzymes which is an indicator of cell viability. Assays were performed upon the samples with exposure of 24 and 48 h after which the assay procedures were carried out. Various concentrations of the samples (250 μg, 100 μg, 50 μg, 10 μg, 1 μg) were prepared by dissolving the required amount in double distilled, filtered PBS through thorough sonication.

### 3.12. Physiochemical Properties

A mathematical equation was used to calculate the density, specific heat, and thermal conductivity of zero-valent NPs (ZVI-NPs) and nanoparticle mixtures in the range of 15 to 45 °C. Additionally, data were collected in the same temperature ranges by combining ZVI-NPs and water in varied ratios of 1:1, 1:2, 1:4, and 1:6 (in mL).

#### Mathematical Formulation

The models of the physiochemical properties were assessed through the following mathematical formulation.

**(a).**  **Density Formulation**

**p = m/V**

where, p is the density (Kgm^−3^), m is mass (Kg), and V is the volume (m^3^) of the ZVI-NPs at varying temperature and water ratios of 1:1, 1:2, 1:4, and 1:6.

**(b).**  **Specific Heat Equation**

**q = mcΔT**

where, q is heat energy (J), m is mass (Kg), c is specific heat capacity (J/(kg °C), and ΔT is change in temperature (°C) of the ZVI-NPs at varying temperature and water ratios of 1:1, 1:2, 1:4, and 1:6.

**(c).**  **Thermal Conductivity Via Heat Transfer**

**K = (QL)/(AΔT)**

where, K is thermal conductivity (W/m·K), Q is amount of heat transferred (J/s), L is distance between the two isothermal planes (m), A is area of the surface (m^2^), and ΔT difference in temperature (°C) of the ZVI-NPs at varying temperature and water ratios of 1:1, 1:2, 1:4, and 1:6.

**(d).**  **Exposure Models**

The simulation of nanomaterial emission and transport in a working or consuming environment as well as in other open-air compartments is done mathematically to estimate exposure (air, water, sediment, soil). The exposure and fate of NPs can be assessed through modelling as well as direct measurements, and numerous particular models have previously been created. The synthesized ZVI-NPs were also modelled for two main exposure models, i.e., worker model and environmental model. All exposure models were analyzed via NanoDesk Platform (http://sudoenanodesk.net/) accessed on 20 February 2023.

In worker model parameters for nanomaterial used, process of the synthesis and room volume were defined. It is possible to mathematically model the fate of the nanoparticle in the environment, which is valuable information. This makes it possible to follow a particle from the moment it enters the environment, whether it arrives through the soil, water, sediment, or air compartment. For this the modelling, parameters are set and synthesized NPs are validated for the environmental exposure.

## 4. Discussion

NPs, which range in size from 1 nm to 100 nm and are invisible to the naked eye, are produced as a result of nanotechnological products. NPs have different chemical and physical properties depending on the material that was used to make them. NPs are divided into many types based on their shape, size, function, chemical and physical properties, and durability [24]. Organic and inorganic NPs are the most common classifications for NPs based on their durability. Liposomes, protein-based NPs, herbal NPs, dendrimers, and other organic NPs are soft, have better dispersibility, and have improved surface characteristics. Inorganic NPs, such as gold NPs, silver NPs, quantum dots, and carbon nanotubes, have a high molecular weight, and are hard in nature. The organic and inorganic NPs are biocompatible, non-toxic, and effective at delivering drugs to their intended targets [25]. Water pollution is one of the global challenges that society must address in the 21st century, aiming to improve water quality and to enhance health impacts of living beings. Many industries generate a large portion of wastewater containing harmful pollutants, especially heavy metals, in the environment, which is becoming a severe problem due to their toxic effects on biological systems of living beings at low concentrations. The discharged heavy metal from industries mainly includes cadmium, lead, mercury, arsenic, and chromium-VI. The toxicity of heavy metals has been a global concern due to their probable role in carcinogenic effects on human beings [26,27,28].

According to contemporary health and environmental problems, heavy metals remain a major source of many diseases as well as the worst impact on habitat in areas near industries. Industrial effluent contains substantial amounts of heavy metals such as cadmium, lead, arsenic, mercury, and chromium-IV, which cause a variety of severe diseases in living beings when deposited untreated into drinking water sources. Heavy metals in industrial wastewater have a variety of negative health impacts on humans, ranging from acute to chronic. When a biological system is exposed to heavy metals for a lengthy period of time, it can cause muscle and neurological degeneration, gastrointestinal, kidney, and immune system dysfunction, skin lesions, vascular damage, and cancer [29]. The scientific research is utilizing the most emerging field, i.e., nanobiotechnology, for the remediation of the heavy metals from industrial wastewater. The industrial wastewater samples were collected from the different tannery industries from Kasur. The collected samples were analyzed for the concentration and detection of heavy metals (cadmium, arsenic, chromium-IV, and lead) in industrial wastewater. The nano bio-system which was used for this research was the biological synthesis of zero-valent iron NPs through seed extract of *Nigella sativa*. The biosystem used in the current research was the seed part of the herbal plant that carries antioxidant compounds. The antioxidant compounds present in the seed extract are unique in their chemical composition, that not only helps in the scavenging of heavy metals from wastewater but also enables the stabilization and reduction of NPs.

The current research is based on the production of ZVI-NPs that utilizes ferric chloride (FeCl_3_) as the precursor. The precursor utilized was prepared in the ratio and molarity. Both seed extract and the precursor FeCl_3_ of specific molarity were mixed in the ratio of 9:1, respectively. After mixing of precursor the color change of precursor from brownish orange to black indicated the synthesis of ZVI-NPs. A certain incubation period was given for the growth of NPs in dark conditions, after that absorption analysis was performed by scanning the wavelength of the ZVI-NPs by UV-Vis Spectrophotometer.

The range for the wavelength absorption by UV-Vis Spectrophotometer of ZVI-NPs was set from 200 nm to 800 nm, of which 250 nm to 370 nm is for ZVI-NPs. The maximum absorption was observed at 274 nm, which confirmed the presence of ZVI-NPs. Scanning electron microscopy coupled with energy-dispersive X-ray spectroscopy (SEM-EDX), and Fourier transform infrared spectroscopy (FTIR) were used to investigate the ZVI-NP composition, shape, elemental constitution, and perspective functional groups, respectively. The synthesized NPs were cylindrical in shape, with a size of 2 nm along with (-OH) hydroxyl, (C-H) alkanes and alkynes N-C, N=C, C-O, =CH functional groups attached to the surface of ZVI-NPs.

The synthesized NPs were then treated with the industrial wastewater at different concentrations: 10 μg, 20 μg, and 30 μg per 100 mL of industrial wastewater in an incubator shaker at 120 rpm for 24 h. After the specific interval of incubation, final concentration of the heavy metals was detected through atomic absorption spectrometer (AAS). The remediated percentage of the heavy metals from the industrial wastewater was then calculated by percentage formula, which has shown that more than 90% of detected heavy metals were removed from the industrial wastewater at 30 μg per 100 mL of ZVI-NPs. The synthesized NPs for the remediation of heavy metals were then subjected to antioxidant, anti-inflammatory, and cytotoxic activities. These activities have enabled us to analyze the biocompatibility of synthesized ZVI-NPs with the biological system of living beings. Thus, the applied analysis for heavy metal removal and the performed activities have given us the pace that the synthesized NPs are suited for with respect to commercial synthesis.

## 5. Conclusions

Nanobiotechnology is one the most emerging fields in science and technology. Seeking the present problems in health and environmental sector, heavy metals remain a major cause of various diseases as well as having the worst impact on habitat in the areas which are surrounded with industries. Industrial wastewater contains heavy metals like cadmium, lead, arsenic, mercury, and chromium-IV in high concentrations that cause multiple lethal diseases in living beings when discharged untreated into drinking water bodies. Previously, heavy metals were removed through conventional methods like flotation, ion exchange mechanism, biosorption, etc., which had some drastic effects on living beings and were not cost effective. The current scientific study describes the synthesis of zero-valent iron NPs (ZVI-NPs) using seed extract of *Nigella sativa* for the indemnification of heavy metals from industrial wastewater. The biologically synthesized zero-valent iron NPs through seed tincture of *Nigella sativa* are unique in remediating heavy metals due to their high efficiency, efficacy with heavy metals, and biocompatibility with living beings. The seed extract of *Nigella sativa* is used for the reduction and stabilization of the ZVI-NPs. The biosynthetic ZVI-NPs have the least cytotoxic effects on biological systems of living beings and are cost effective. The synthesized ZVI-NPs will help in society in solving both health and environmental issues caused by the influence of heavy metals by treating wastewater at the industrial level.

## Figures and Tables

**Table 1:** Initial concentrations of heavy metals by Atomic Absorption Spectrometry (AAS) in ppm and permissible values of heavy metals in industrial waste water by WHO.

Detected Heavy Metals	Cadmium (Cd) (ppm)	Mercury(Hg) (ppm)	Arsenic (As) (ppm)	Chromium-IV (Cr-IV) (ppm)	Lead (Pb) (ppm)
Conc. of heavy metals by WHO	0.003	0.001	0.01	0.05	0.01
S-1	29.6	N.D	0.05	N.D	2.4
S-2	52	N.D	0.08	1316.5	5
S-3	76.4	N.D	0.07	N.D	7.4
S-4	100.2	N.D	0.06	0.45	10.4
S-5	124.2	N.D	0.08	442.5	12.8

**Table 2:** Phytochemically screened compounds present in the seed tincture of *Nigella sativa*.

Phytochemical Test		Screened Phytochemical	Interference	Result
1	Wagner’s Test	Alkaloids	Appearance of reddish brown color with Wagner’s reagent	Present
2	Foam Test	Saponins	Formation of stable foam	Present
3	Ferric Chloride Test	Phenols	Indication of blue green color with ferric chloride	Present
4	Braymer’s Test	Tannins	Formation of green precipitates	Present
5	Salkowski’s Test	Terpenoids	Appearance of yellow color	Present
6	Bontrager’s Test	Quinones	Occurrence of red color in alkaline phase	Absent
7	Keller–Killani’s Test	Cardiac Glycosides	Formation of pink to blood red coloration	Present
8	Glycosides Test	Glycosides	Indication of pink color	Absent
9	Alkaline Reagent Test	Flavonoids	Formation of yellow color which becomes colorless on addition of acid	Present
10	Precipitate Test	Phlobatannins	Appearance of red precipitates	Absent

**Table 3:** Final concentrations of heavy metals detected after treatment of industrial wastewater by ZVI-NPs.

SR#.	Cr-VI (ppm)	Pb (ppm)	As (ppm)	Cd (ppm)
IS-1 (T-1) (10 μg)	Nil	0.6	0.74	9.6
IS-1 (T-2) (20 μg)	Nil	0.4	0.39	6.74
IS-1 (T-3) (30 μg)	Nil	0.02	0.2	2.06
IS-2 (T-1) (10 μg)	186.8	3.01	3.39	15.01
IS-2 (T-2) (20 μg)	46.8	1	2.59	10.2
IS-2 (T-3) (30 μg)	41	0.5	1.05	8.09
IS-3 (T-1) (10 μg)	Nil	2.5	7.02	9.44
IS-3 (T-2) (20 μg)	Nil	1.03	5.42	4.05
IS-3 (T-3) (30 μg)	Nil	0.42	2.55	2.29
IS-4 (T-1) (10 μg)	20	3.58	6.45	29.22
IS-4 (T-2) (20 μg)	13.53	2.07	4.44	13.01
IS-4 (T-3) (30 μg)	8.41	1.6	2.9	1.84
IS-5 (T-1) (10 μg)	102	4.5	5.58	29.64
IS-5 (T-2) (20 μg)	84.8	3	3.48	18.3
IS-5 (T-3) (30 μg)	50.9	1.26	1.02	5.83

**Table 4:** Statistical analysis of % inhibition in protein denaturation showing statistically significant (*p* < 0.01) results.

% Inhibition of Protein Denaturation Anti-Inflammatory Activity (660 nm)												
Blank: Distilled Water	Control Aspirin: 2.370 Abs				Seed Extract of *Nigella sativa*				ZVI-NPs			
Conc.	R1	R2	R3	Mean	R1	R2	R3	Mean	R1	R2	R3	Mean
100 μg/mL	50.55	50.6	50.7	50.61	55.7	55.8	55.9	55.8	60.3	60.4	60.4	60.3
200 μg/mL	65.3	65.4	65.2	65.3	68.9	68.9	69	68.9	84.4	84.5	84.4	84.4
300 μg/mL	72.5	72.7	72.4	72.5	74.1	74	74	74	85.1	84.9	84.8	84.9
400 μg/mL	80.56	80.4	80.7	80.55	75	75.1	75	75	89.7	89.7	89.8	89.7
500 μg/mL	85.7	85.6	85.4	85.52	75.7	75.8	75.7	75.7	96	96.1	96.16	96

**Table 5:** Percentage free radical scavenging activity and calculated IC50 values of standard (ascorbic acid), seed extract of *Nigella sativa,* and ZVI-NPs.

Anti-oxidant Activity (517 nm)						
Concentration (μg/mL)	% Free Radical Scavenging			IC50		
	Ascorbic Acid	Seed Extract	ZVI-NPs	Ascorbic Acid	Seed Extract	ZVI-NPs
200	40.5	34.2	50.6	596.7	387.56	194.77
400	50.9	46	69.1			
600	65.4	45.9	78.7			
800	70.3	49.1	87.3			
1000	79.5	61.42	87.7			

**Table 6:** Calculated IC50 values of U87-MG and HEK cell lines.

IC50 Values				
Concentration	U87-MG Cell Line 24 Hours	48 h	HEK Cell Line 24 Hours	48 h
250	45.15166	39.70977	54.03226	53.87948
100	74.33166	46.19977	74.03226	75.24948
50	79.08166	52.39977	76.03226	79.98948
10	88.93166	94.13977	91.03226	91.27948
1	92.76166	98.31977	92.03226	91.86948

**Table 7:** Morphology of U87-MG and HEK cell lines after 24 h and comparison with the control.

Cell Morphology						
Positive Control		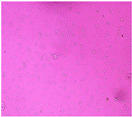	Solvent Control	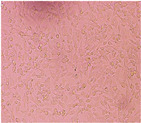	MTT Treated Cell	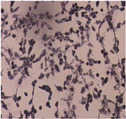
Concentration		250	100	50	10	1
U87-MG Cell Line	After 24 Hours	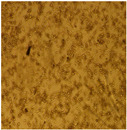	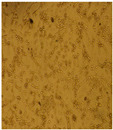	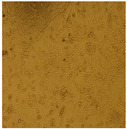	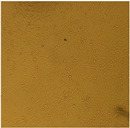	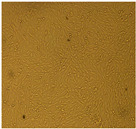
HEK Cell Lines		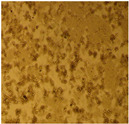	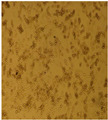	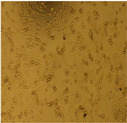	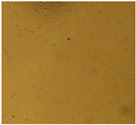	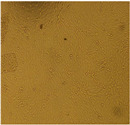

**Table 8:** Variation in density (Kg/m^3^) as a function of solvent content and temperature.

Temperature (°C)	ρ^1b^	ρ^2b^	ρ^3b^	ρ^4b^
15	80	133.33	142.28	155.21
20	74.07	287.43	298.45	301.25
25	66.67	365.28	355.82	345.28
30	57.14	297.45	288.58	272.65
35	68.96	478.58	468.23	488.12
40	64.51	582.24	525.54	565.45

ρ^1b^ = density of zero-valent iron NPs (ZVI-NPs); ρ^2b^, ρ^3b^, ρ^4b^ = density after mixing NPs and water in the ratio 1:2, 1:4, 1:6 (in mL).

**Table 9:** Variation of the nanofluid’s specific heat (J/K/mol) with temperature and the volume of solvents.

Temperature (°C)	ρC^p^	ρC^p2^	ρC^p3^	ρC^p4^
15	320	400	500	600
20	160	200	250	300
25	480	600	750	900
30	640	800	1000	1200
35	960	1200	1500	1800
40	800	1000	1250	1500

ρC^p1^ = Specific heat capacity of ZVI-NPs; ρC^p2^, ρC^p3^, ρC^p4^ = Specific heat of ZVI-NPS after mixing NPs and water in 1:2, 1:4, 1:6 (in mL).

**Table 10:** Shows thermal conductivity (Knf) varies with temperature and solvent concentration.

Temperature (°C)	Knf^1^	Knf^2^	Knf^3^	Knf^4^
15	390.65	1132.31	1145.18	1158.25
20	188.63	915.38	928.67	945.14
25	598.16	2791.94	2787.96	2777.51
30	388.21	2526.11	2537.43	2558.28
35	501.98	4354.68	4342.24	4377.45
40	102.4	5562.8	5505.21	5578.49

Knf^1^ = Thermal conductivity of ZVI-NPs; Knf^2^, Knf^3^, Knf^4^ = thermal conductivity of ZVI-NPs after mixing NPs and water in 1:2, 1:4, 1:6 (in mL).

## Data Availability

Not Applicable.
